# Accuracy of Different COVID-19 Severity Scores in ICU Transfer Prediction and Mortality Prediction Among Inpatients at Lebanese American University Medical Center-Rizk Hospital (LAUMC-RH)

**DOI:** 10.7759/cureus.95269

**Published:** 2025-10-23

**Authors:** Evelyne Towair, Zeina Al Achkar, Romanos Haykal, Ahmad Kassar, Eric Bou Karam, Pascale Salameh, Toufic Chaaban

**Affiliations:** 1 Internal Medicine, Lebanese American University Medical Center-Rizk Hospital, Beirut, LBN; 2 Pulmonary and Critical Care Medicine, Lebanese American University Medical Center-Rizk Hospital, Beirut, LBN; 3 Primary Care and Population Health, University of Nicosia Medical School, Nicosia, CYP; 4 Public Health, Institut National de Santé Publique, Epidémiologie Clinique et Toxicologie (INSPECT-LB), Beirut, LBN; 5 Pharmacy, Lebanese University, Beirut, LBN; 6 School of Medicine, Lebanese American University, Beirut, LBN

**Keywords:** covid-19, icu admission, invasive mechanical ventilation, lebanon, mortality

## Abstract

Background: The healthcare system has been immensely drained by the COVID-19 pandemic. This required the use of multiple triage methods to allow better management of patients’ care and resource distribution. Multiple severity scores have been proposed as predictors of mortality, ICU stay, and the need for intubation among COVID-19 patients. However, the efficiency of these scores in the Middle East and North Africa (MENA) region, specifically Lebanon, hasn’t been properly assessed.

Objectives: This study aims to evaluate the accuracy of the five COVID-19 severity scores: COVIC, CURB-65, ISARIC4C, COVID-GRAM, and CALL in foreseeing mortality, critical illness, and intubation needs among patients presenting to the emergency departments of the Lebanese American University Medical Center-Rizk Hospital (LAUMC-RH).

Methods: This is a retrospective study analyzing 520 patients who tested positive for COVID-19 between August 2020 and June 2021. Data was extracted from the electronic medical records, and statistical analysis was done to assess the predictive performance of each score.

Results: The in-hospital mortality was 12.7%. All studied scores showed correlation with mortality; however, ISARIC4C and COVIC had superior sensitivity compared to the other scores. When studying the need for ICU admission, COVID-GRAM had the best predictive accuracy, while COVIC was able to accurately predict the need for intubation. Furthermore, CURB-65 had the lowest specificity in all outcomes.

Conclusion: Despite the ability of all five scores in predicting the severity of COVID-19, their predictive accuracy varied. Taking into consideration this being a single-center study, further multicenter validation studies in the MENA region are needed to define the optimal triaging model for COVID-19 infection or any future respiratory pandemic.

## Introduction

The novel coronavirus known as SARS-COV-2 emerged at the end of 2019 in Wuhan, China. The WHO declared the novel coronavirus outbreak a pandemic on March 11, 2020 [1]. More than 775 million cases of COVID-19 infections were confirmed globally, with around seven million deaths that were attributed to the virus, according to the World Health Organization (WHO) cases report [2]. A retrospective study done in Wuhan in 2020 showed that 42% of patients with COVID pneumonia developed acute respiratory distress syndrome (ARDS), and 61-81% of them required intensive care [3]. Abate et al. conducted a meta-analysis that included 37 articles with 24,983 participants and showed that the mortality rate of ICU patients with coronavirus reached 39% [4]. The incidence of ARDS and mortality changed later with the development of new variants, widespread vaccination, and baseline immunity by infection, with the incidence of ARDS among all COVID-related deaths being 10.8% [5].

The pandemic has globally affected clinical and hospital practice, placing significant strain on the healthcare system [6,7]. Low- and middle-income countries are not exempted, especially with the scarcity of intensive care units, the limited number of mechanical ventilators, and the prevalence of comorbidities [8]. In such a pandemic, therapeutic decisions, including admitting patients to critical care units and providing other aggressive treatments (mechanical ventilation, high flow oxygen therapy), are important decisions to be made by healthcare providers. Thus, appropriate triage of patients during epidemics is crucial for the appropriate use of resources and workforces [9].

 Many scores have been used during the COVID-19 epidemic to help with the triage of patients, predicting the prognosis, and the level of care needed. The COVIC score, developed by Heo et al., evaluated the risk of critical illness in COVID patients based on seven variables [10]. The second score is the CURB-65, although it was validated previously in patients presenting to the ED with community-acquired pneumonia; however, this score proved to be effective as well for COVID pneumonia critical illness risk assessment [11]. Lastly, Liang et al. created a score named COVID-GRAM, which was also used to predict critical illness [12]. Two scores were used to predict in-hospital mortality: the ISARIC4C mortality score, consisting of eight variables, and the CALL score, developed by Grifoni et al., with only four variables [13,14]. 

Several studies compared the accuracy and predictive ability of these different scores [15,16]. These scores were mainly derived and validated from populations in developed countries, but not in the Middle East and North Africa (MENA) region, and Lebanon in particular.

Our study aims to evaluate these scores in predicting mortality, the need for mechanical ventilation, critical illness, and progression risk within a cohort of patients with COVID-19 presenting to the Emergency Department of a tertiary care centre in Lebanon. This research will contribute to the management of patients with COVID-ARDS and potentially provide insight into their application in other respiratory pathogens.

## Materials and methods

Study design

The aim of this retrospective study is to evaluate the accuracy of the different COVID-19 severity scores in predicting critical care unit admission as a primary outcome in COVID-19 patients presenting to the emergency department of LAUMC-Rizk Hospital. In-hospital mortality, mechanical ventilation needs, and 28-day mortality were the main secondary outcomes. The study was approved by the Institutional Board Review of the Lebanese American University.

Patients were included if they were 18 years old or older and had a documented positive polymerase chain reaction (PCR) test for SARS-CoV-2 before or during the first 48 hours of ED presentation. Data collection was done retrospectively from the electronic charts between August 2020 and June 2021.

Exclusion criteria included patients transferred from other hospitals with more than 24 hours stay there, those who are found to be COVID-positive after 48 hours of admission, patients needing more than 10 liters of oxygen on admission, and those who are on noninvasive ventilation at home.

This population was followed longitudinally by reviewing the clinical archives and medical records from the time of the first presentation until the date of the study, following up on disease progression, whether those patients required critical care, mechanical ventilation, as well as death related to COVID pneumonia in-hospital or within 28 days of discharge. 

Statistical analysis

The collected data were analyzed using IBM SPSS Statistics for Windows, Version 28 (Released 2021; IBM Corp., Armonk, New York, United States). For descriptive analysis, frequency and percentage were used for categorical variables, and mean and standard deviation for quantitative variables. The distribution of these variables was considered normal using visual inspection of the histogram, while the skewness and kurtosis were lower than one. These conditions are considered compatible with normality with a sample size higher than 300. For the bivariate analysis of continuous variables, the Student’s T-test was used to compare the means between two groups and analysis of variance (ANOVA) to compare between three groups or more, after checking for homogeneity of variances using Levene’s test. In case the variances are not homogeneous, the corrected T-Test and the Kruskal-Wallis test were used, respectively. For categorical variables, the Chi-squared test was used, and Fisher's exact test was used in cases of expected values less than five. Moreover, receiver operating characteristic (ROC) curves were used to find the cutoff values of scales that could better predict mortality related to COVID; respective areas under the curve, sensitivity, and specificity values were calculated. 

## Results

Study population and baseline characteristics

A total of 520 patients admitted to LAUMC-Rizk Hospital with confirmed COVID-19 infection were included in this study. As evident in Table 1, the age range of the patients was between 21 and 97, with a mean of 63. The male gender was more predominant than the female, being 68% of the studied population. On average, the patients included have at least one comorbidity. The most common presenting symptom across the selected population was dyspnea (62.6%). 

**Table 1:** Patient’s characteristics and presenting symptoms COPD: chronic obstructive pulmonary disease; CKD: chronic kidney disease; CAD: coronary artery disease

	Total (n = 520)/percentage
Mean age (in years)	63/12%
Gender	
Male	355/68%
Female	165
Mean number of comorbidities	1
% of comorbidities	30% one comorbidity; 15% two comorbidities; 5.3% three comorbidities; 2.2% more than three comorbidities
Comorbidities	
Hypertension	256 /49%
Diabetes	160/30.7%
COPD	16/3%
CKD	18/3.5%
Liver disease	5/0.9%
At least one cardiac comorbidity, CAD	130/25%: 66/12.6%
Neurologic disease	39/7.5%
Malignancy solid hematologic	40/7.6%, 27/5%, 11/2%
Presenting symptoms	
Dyspnea	326/62.6%
Fever	302/58%
Hemoptysis	3/0.57%
Altered mental status	69/13%

Mortality

The in-hospital mortality rate was found to be 12.7%, 66 out of 520 patients. When assessing the correlation between the incidence of death and the multiple cofactors, no significant correlation was found between death and both genders and having infiltrates on imaging upon presentation, with p-values of 0.765 and 0.769, respectively. Regarding the symptoms of those patients, a significant correlation between having an altered mental status on presentation and a higher risk of mortality was seen (p < 0.001), as well as fever (p = 0.05) (Appendix Table 8). However, dyspnea on presentation and hemoptysis were not associated with mortality. Moreover, some comorbidities were significantly associated with a higher mortality rate, such as hypertension (p < 0.001), history of cardiac illness (p < 0.001), mainly those with multiple cardiac comorbidities, and history of liver disease (Appendix Table 8). No significant relationship was found between mortality and other comorbidities.

As seen in Table 2, there was a significant correlation between high mortality rate and age (mean age 73.33; SD, 14.84; p < 0.001), number of comorbidities, (mean number of comorbidities, 1.48; SD, 1.4; p < 0.001), having a disturbed MAP (mean MAP, 89.2; SD, 16.6; p = 0.009), and SpO2 mean value of 89.2 (SD, 13.4; p < 0.001), and having a high percentage of lung involvement (mean percentage, 64.2%; SD, 25.3%; p < 0.001).

**Table 2:** Association between means of variables, scores, and mortality MAP: mean arterial pressure; SpO2: oxygen saturation; CRP: C-reactive protein *: means the correlation is significant at p < 0.05

	Mortality	Mean	Standard deviation	p-value
Age (in years)	Yes	73.33	14.84	<0.001*
	No	61.84	15.394	
Number of comorbidities	Yes	1.48	1.417	<0.001*
	No	0.79	0.964	
MAP	Yes	89.2	16.6	0.009*
	No	93.5	13.5	
SpO2	Yes	86.5	13.4	<0.001*
	No	92.7	7.3	
Liters of O2	Yes	3.1	5.42	0.3
	No	1.5	5.77	
% of lung involvement	Yes	64.2%	25.3%	<0.001*
	No	43.5%	21.6%	
COVIC score	Yes	27.7	5.98	<0.001*
	No	16.4	6.21	
CURB-65	Yes	2.09	1.048	<0.001*
	No	1.06	1.026	
ISARIC4C	Yes	11.52	3.55	<0.001*
	No	7.16	4.0	
COVID-GRAM	Yes	166.7	58.3	0.038*
	No	137.0	36.3	
CALL score	Yes	10.75	1.85	<0.001*
	No	9.03	2.31	

When assessing the significance of the various COVID scores in predicting mortality, all the included scores showed a significant correlation with predicting mortality; elevated COVIC score with the mean score being around 27.7 with SD of 5.98 (p < 0.001), CURB-65 with a mean of 2.09 (SD, 1.048; p < 0.001); ISARIC4C with a mean of 11.52 (SD, 3.55; p < 0.001), and CALL-score with a mean of 10.75 (SD, 1.85; p < 0.001), as demonstrated in Table 2.

Need for intubation 

A total of 76 patients (14.6%) required intubation (Appendix Table 9). There was a significant correlation between having dyspnea (p = 0.008) and altered mental status (p < 0.001) and requiring intubation. As for their comorbidities, there was a significant correlation between intubation and those with a medical history of cardiac disease (p = 0.004), mainly those with multiple cardiac diseases, diabetes (p = 0.02), and hypertension (p < 0.001). However, there was no statistical significance between the need for intubation and gender (p = 0.406), having a history of chronic pulmonary disease (p = 0.380), and having infiltrates on imaging (p = 0.446). 

When conducting a t-test study for intubation. We noticed that there was a significant correlation between patients’ age and their need for intubation (the mean age was 68.7 with SD ± 14.7, with a p-value = 0.001); there was also a significant correlation with the number of comorbidities (mean number, 1.28; SD ± 1.33; p = 0.002) (Table 3). 

**Table 3:** Association between means of variables and scores with the need for intubation MAP: mean arterial pressure; SpO2: peripheral oxygen saturation; CRP: C-reactive protein *: means the correlation is significant at the p < 0.05 level

	Intubation	Mean	Standard deviation	p-value
Age (in years)	Yes	68.7	14.7	0.001*
	No	62.3	15.7	
Number of comorbidities	Yes	1.28	1.33	0.002*
	No	0.81	0.98	
MAP	Yes	17.37	1.99	0.091*
	No	13.3	0.63	
SpO2	Yes	84.9	13.6	<0.001*
	No	93.14	6.7	
Liters of O2	Yes	4.5	8.7	0.001*
	No	1.29	4.9	
CRP	Yes	11.9	9.2	0.031*
	No	9.1	13.6	
% of lung involvement	Yes	63.8%	24.5%	<0.001*
	No	42.6%	21.1%	
COVIC score	Yes	22.5	5.6	<0.001*
	No	16.3	6.2	
CURB-65	Yes	1.99	1.12	<0.001*
	No	1.05	1.019	
ISARIC4C	Yes	10.9	4.06	<0.001*
	No	7.15	1.12	
COVID-GRAM	Yes	168.7	56.1	0.025*
	No	135	34.7	
CALL score	Yes	10.4	2.02	<0.001*
	No	9.07	2.32	

Regarding the scores, all the studied scores showed a significant correlation with their ability to predict the need for intubation. COVIC score had a mean of 22.5 with SD ± 5.6 and p = 0.00; CURB-65 score had a mean value of 1.99 with SD ± 1.12 and p = 0.00; ISARIC4C mean value of 10.9 with SD ± 4.06 and p = 0.00; COVID-GRAM had a mean value of 168.7 with SD ± 56.1 and p-value = 0.025; and CALL-score had a mean value of 10.4 with SD ± 2.02 and p < 0.001 (Table 3). 

Duration of ICU stay

As for the need for ICU stay, 148 patients (28.5%) required ICU admission. Our bivariate analysis, in the appendix, showed a statistical significance between needing ICU admission and having an altered mental status (p < 0.001) and dyspnea (p < 0.001) on presentation. 

Regarding the link between the various scores and their correlation with the duration of ICU stay (Table 4), there was only a significant correlation with CURB-65 with a mean value of 21.5 (SD ± 1.17; p = 0.049) and CALL score with a mean value of 10.26 in comparison to 8.9 among those who didn’t require ICU stay (SD ± 1.93; p = 0.12). It was remarkable that COVID-GRAM and COVIC, which are the scores that were used to assess the risk of critical illness and ICU admission, did not significantly correlate with the risk of ICU stay in our population (p = 0.433 and 0.838, respectively).

**Table 4:** Association between the means of different variables, scores, and critical care admission MAP: mean arterial pressure; SpO2: peripheral oxygen saturation; CRP: C-reactive protein *: means the correlation is significant at the p < 0.05 level

	ICU stay	N	Mean	Standard deviation	p-value
Age (in years)	Yes	148	67.84	15.17	0.398
	No	371	61.49	15.67	
Number of comorbidities	Yes	147	1.29	1.2	<0.001*
	No	370	0.72	0.9	
MAP	Yes	148	91.13	15.9	0.002*
	No	372	93.73	13.1	
SpO2	Yes	148	88.11	11.5	<0.001*
	No	372	93.47	6.5	
Liters of O2	Yes	148	4.4	9.7	<0.001*
	No	372	0.687	2.2	
CRP	Yes	139	11.68	8.9	0.643
	No	351	8.7	14.3	
% of lung involvement	Yes	67	61.8%	25.8	<0.001*
	No	201	40.35%	19.04	
COVIC score	Yes	148	21.5	5.88	0.838
	No	372	15.5	5.95	
CURB-65	Yes	146	1.73	1.17	0.049*
	No	368	0.98	0.996	
ISARIC4C	Yes	137	10.2	3.93	0.469
	No	346	6.74	3.89	
COVID-GRAM	Yes	29	161.45	51.2	0.433
	No	54	132.1	32.9	
CALL-score	Yes	70	10.26	1.93	0.12*
	No	176	8.9	2.36	

Correlation of scores with different variables

A Pearson correlation coefficient was computed to assess the linear relationship between the scores we used in our study and the different variables. In summary (Table 5), the patient’s age correlated positively with all the scores we used, and this correlation was the highest with the ISARIC4C score. The number of comorbidities was moderately correlated positively with ISARIC4C, COVID-GRAM, and CALL score. And what is also remarkable is that the oxygen saturation was negatively correlated to COVIC, ISARIC4C, and COVID-GRAM scores, and this correlation was low.

**Table 5:** Correlation of the scores with different variables SBP: systolic blood pressure; DBP: diastolic blood pressure; MAP: mean arterial pressure; RR: respiratory rate; SpO2: peripheral oxygen saturation; CRP: C-reactive protein *: means the correlation is significant at the p < 0.05 level. **: means the correlation is significant at the p < 0.01 level

Variable	COVIC	CURB65	ISARIC4C	COVID-GRAM	CALL score
Age	.49** (p < .001)	.66** (p < .001)	.79** (p < .001)	.31** (p = .004)	.58** (p < .001)
Temperature	.07 (p = .097)	−.17** (p < .001)	−.15** (p = .001)	−.32** (p = .002)	−.16** (p = .008)
Number of comorbidities	.33** (p < .001)	.43** (p < .001)	.56** (p < .001)	.58** (p < .001)	.62** (p < .001)
SBP	.03 (p = .441)	−.20** (p < .001)	.016 (p = .720)	−.18 (p = .096)	−.06 (p = .324)
DBP	−.09* (p = .026)	−.41** (p < .001)	−.17** (p < .001)	−.34** (p = .002)	−.19** (p = .003)
MAP	−.03 (p = .391)	−.35** (p < .001)	−.09* (p = .036)	−.29** (p = .008)	−.14* (p = .022)
RR	.04 (p = .346)	.17** (p < .001)	.08* (p = .050)	.25* (p = .022)	−.05 (p = .427)
SpO₂	−.31** (p < .001)	−.28** (p < .001)	−.36** (p < .001)	−.30** (p = .005)	−.23** (p < .001)
Liters	.10* (p = .014)	.06 (p = .132)	.06 (p = .169)	.27* (p = .011)	.004 (p = .921)
CRP	.19** (p < .001)	.03 (p = .508)	.18** (p < .001)	.38** (p < .001)	.09 (p = .146)
Absolute lymphocyte count	−.09* (p = .027)	−.03 (p = .427)	−.06 (p = .133)	−.21 (p = .051)	−.18** (p = .005)
% of lung involvement	.33** (p < .001)	.20** (p = .001)	.25** (p < .001)	.39 (p = .085)	.25** (p = .004)
Length of stay	.19** (p < .001)	.14** (p = .001)	.26** (p < .001)	.16 (p = .141)	.16* (p = .010)
Length of ICU stay	.20** (p = .002)	.14* (p = .028)	.20** (p = .004)	−.00 (p = .965)	−.02 (p = .816)

Scores

In our statistical analysis, we compared the mean of the scores that we used in regard to the outcomes that we looked for. The means of each score and their 95% confidence interval concerning each outcome are summarized in Table 6. The bars of the estimated means for the assessed scores are available in the appendix. 

**Table 6:** Comparison between the means of the scores and the outcomes ICU: intensive care unit

Score	CURB-65	ISARIC4C	COVID-GRAM	COVIC	CALL
Death-no	1.2 (0.6-1.6)	8 (7-10)	150 (140-170)	17 (12-21)	9.5 (9-11)
Death-yes	2 (0.8-3.2)	10.5 (7-14)	160 (130-200)	19 (14-24)	11.5 (9-13.5)
ICU admission-no	1.2 (0.2-4)	7.3 (3-11)	150 (120-180)	14 (8-19)	9.5 (9-11)
ICU admission-yes	1.8 (1.1-2.5)	11 (8-13)	160 (140-180)	21 (18-24)	10.2 (9-11)
Intubation-no	1.6 (0.8-2.5)	9.5 (6.5-12.5)	150 (125-170)	17.5 (14-21)	10 (9-11.5)
Intubation-yes	1.4 (0.8-2.1)	9.5 (7.5-11.8)	170 (150-180)	21 (18-24)	11 (9-12.5)

**Figure 1:**
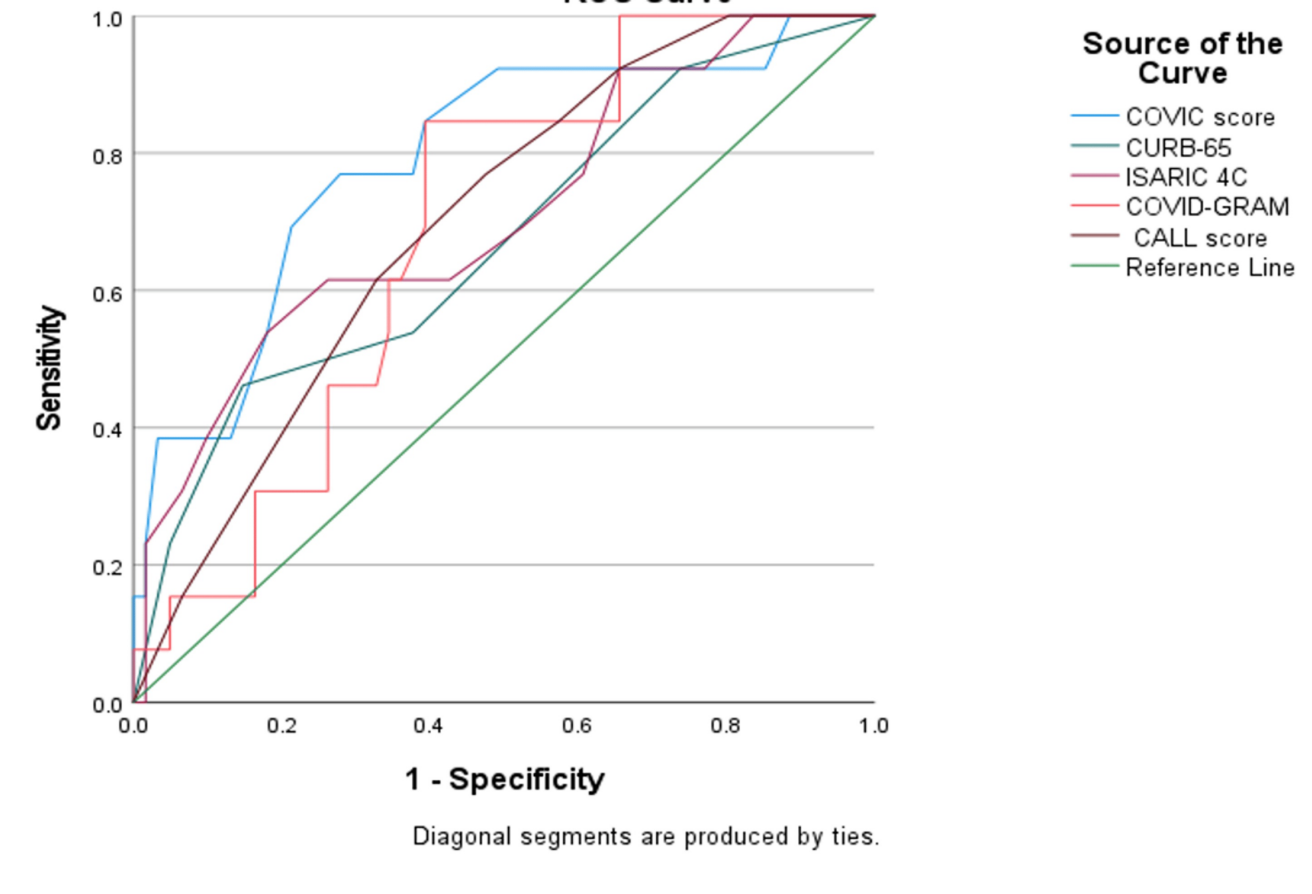
Mortality ROC curve ROC: receiver operating characteristic

**Figure 2:**
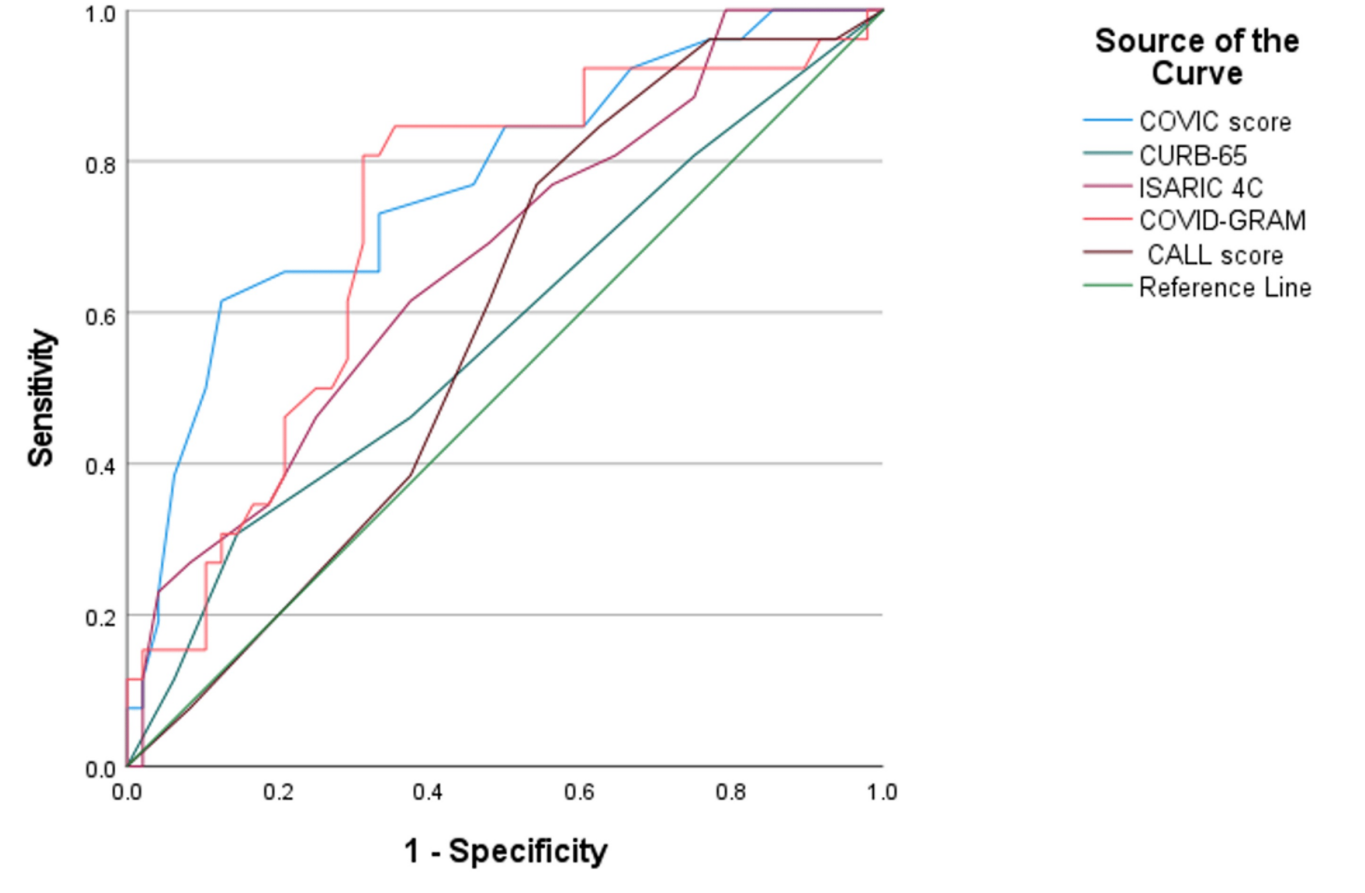
Critical care ROC curve ROC: receiver operating characteristic

ROC curves were used to assess the predictive efficacy of the scores and each outcome in the appendix. A significant relationship was found between all the scores and the mortality (Figure 1), as well as all the scores and needs for critical care except the CALL score (p < 0.05) (Figure 2). However, regarding the need for mechanical ventilation, only the COVIC score and COVID-GRAM had a significant relationship (p =< 0.05). 

Performance in predicting mortality was not optimal in the scores that we assessed, with an AUC of 0.791, 0.67, 0.709, 0.688, and 0.699 from the COVIC score, CURB-65, ISARIC4C, COVID-GRAM, and CALL score, respectively. The sensitivity was high for the COVIC score, and COVID-GRAM was high, reaching 84.6% for a score of >18.5 and 84.6% for a score of >145.5, respectively. However, the specificity reached 61% and 60%, respectively. The sensitivity and the specificity were not high for the rest of the scores (Table 7).

**Table 7:** Performance, sensitivity, and specificity of the score AUC: area under the curve; CI: confidence interval; ICU stay: intensive care unit stay *: means the correlation is significant at the p < 0.05 level

Outcome & score	AUC	95% CI	Sensitivity (%)	Specificity (%)	p-value
Mortality-COVIC score > 18.5	0.791	(0.651-0.932)	84.6	61	0.001*
Mortality-CURB-65 > 1.5	0.670	(0.502-0.893)	53.8	63	0.055*
Mortality-ISARIC4C > 10.5	0.709	(0.547-0.872)	61.5	74	0.018*
Mortality-COVID-GRAM > 145.5	0.688	(0.554-0.822)	84.6	60	0.034*
Mortality-CALL score > 11.5	0.699	(0.558-0.839)	61.5	68.2	0.025*
ICU stay-COVIC score > 18.5	0.772	(0.657-0.886)	73	67	<0.001*
ICU stay-CURB-65 > 0.5	0.573	(0.433-0.796)	80.8	25	0.300
ICU stay-ISARIC4C > 9.5	0.669	(0.542-0.796)	61.5	63	0.017*
ICU stay-COVID-GRAM > 138.5	0.719	(0.596-0.843)	84.6	62.5	0.002*
ICU stay-CALL score > 10.5	0.587	(0.456-0.717)	61.5	52	0.221
Intubation-COVIC score > 18.5	0.791	(0.659-0.924)	81.3	60	<0.001*
Intubation-CURB-65 > 0.5	0.564	(0.396-0.732)	81	24	0.435
Intubation-ISARIC4C > 8.5	0.637	(0.486-0.788)	62.5	47	0.094
Intubation-COVID-GRAM > 145.5	0.715	(0.591-0.839)	81	60.3	0.009*
Intubation-CALL score > 10.5	0.587	(0.434-0.740)	62.5	64	0.291

The performance in predicting the need for critical care was high for all scores, with the AUC of 0.772, 0.573, 0.669, 0.719, and 0.587 for the COVIC score, CURB-65, ISARIC4C, COVID-GRAM, and CALL score, respectively. The sensitivity was the highest among the CURB-65 and COVID-GRAM scores (80.8% and 84.6%, respectively), with a corresponding sensitivity of 25% and 62.5%, and the sensitivity and specificity of the rest of the scores were not as high (Table 7).

**Figure 3:**
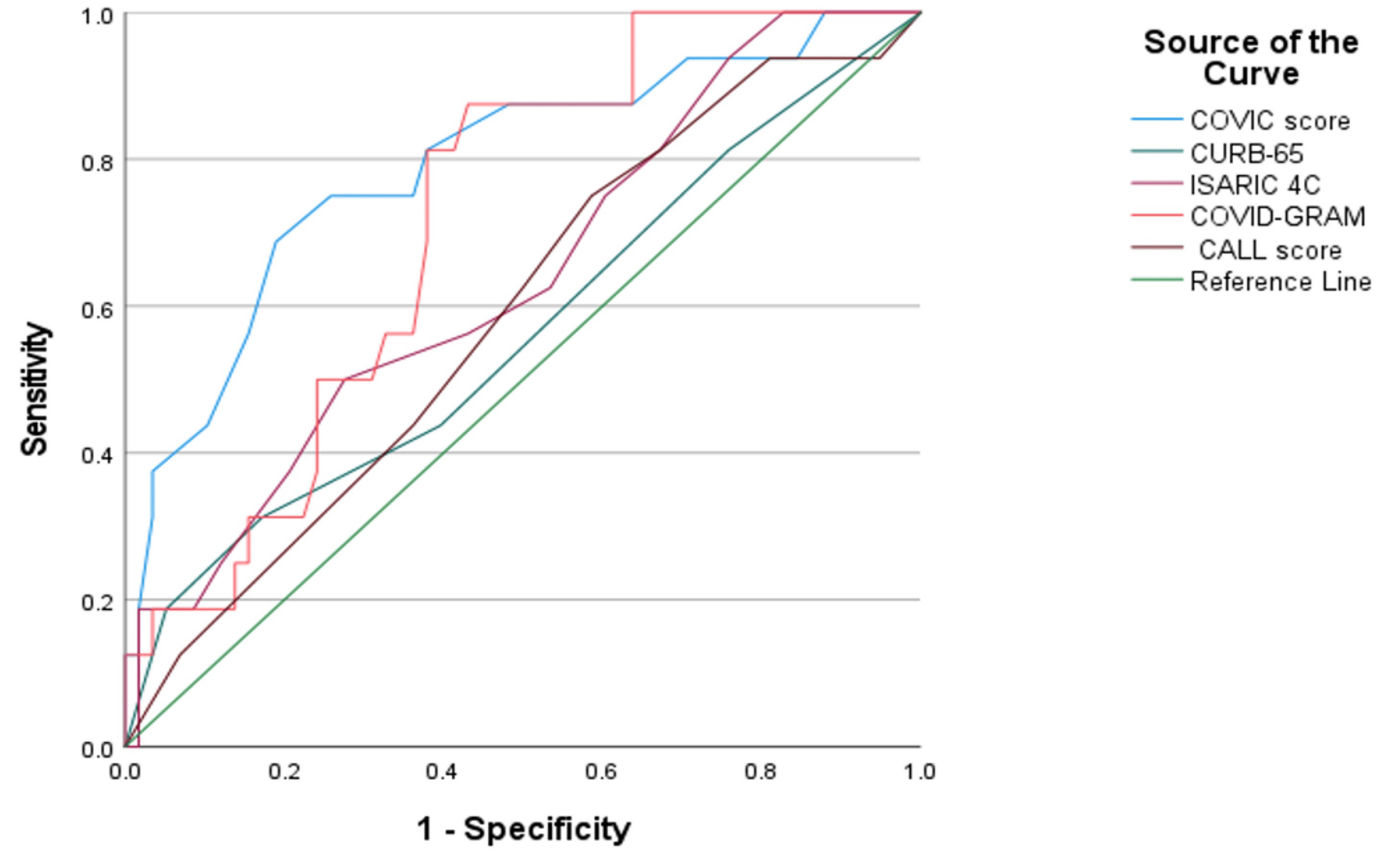
Intubation ROC curve ROC: receiver operating characteristic

Furthermore, the performance of the scores in predicting the need for intubation was high for COVIC and COVID-GRAM scores, with an AUC of 0.791 and 0.715, respectively. The sensitivity was high and almost similar for both scores, reaching 81.3% for COVIC and 81% for COVID-GRAM, as well as for the specificity, reaching 60% and 60.3%, respectively (Table 7 and Figure 3).

## Discussion

Effective triage of patients with COVID-19 infection has an important and vital role in the allocation of medical treatment and resource control, especially during pandemics. Multiple efforts have been made to try and establish stratification scores or identify preexisting models to predict adverse outcomes based on both clinical features and laboratory markers. Furthermore, having a score that allows physicians to predict patients’ need for further mechanical ventilation and critical care, and to predict the mortality risk, is helpful in providing the needed care. However, there was significant heterogeneity in the variables used and statistical methods employed, making it challenging to compare the scores’ performance. For this, the following study was designed to compare five scores that can help predict mortality and overall hospital outcomes in patients diagnosed with COVID-19.

Mortality

The in-hospital mortality in our study was reported to be 12.7%. This finding has also been reported in other published studies, like the ones published by Alimohamadi et al, while others documented a higher rate reaching up to 19% [17]. Despite other studies showing an impact of gender and other comorbidities on mortality rates, our study didn’t report any significant correlation with gender [18,19]. However, there was a higher mortality rate among those with a history of liver disease. In accordance with other studies, there was a higher mortality rate among those who had dyspnea on presentation [19]. Other studies showed a correlation with having other symptoms on presentation [20]. When looking at radiological findings, there was a higher mortality among those with higher lung involvement on CT, which was also documented in other studies [21].

In our study, the scores that were included were able to successfully predict mortality. ISARIC4C and CURB-65 are the two scores that were initially used to predict mortality in COVID patients. According to our findings, an ISARIC4C score of more than 10.4, which represents a high-risk score, is more sensitive and specific (61.5% and 74%, respectively) than CURB-65 in predicting mortality, which had a sensitivity of 53.8% and a specificity of 63% with a score of more than 1.5. Moreover, the sensitivity of the COVIC score and COVID-GRAM was higher in predicting mortality at 84.6% for both; however, the specificity of those two scores was not as high, reaching 61% for a COVIC score of more than 18.5 and a COVID-GRAM score of more than 145.5.

When discussing CURB-65, in a study done by Na et al., CURB-65 was a poor predictor of mortality [22]. But other studies showed the effectiveness of this score in assessing mortality in different countries such as Italy, Saudi Arabia, and Iran, among COVID-19 patients. In a study done by Khari et al., comparing the accuracy of CURB-65 and other scores, it showed that the estimated sensitivity of CURB-65 ranged between 96.59% and 87.50% [23-25]. However, when they assessed specificity, CURB-65 had a specificity of 6.75% [23-25]. Moreover, in a study done by Citu et al., despite performing well in predicting mortality due to COVID-19 when compared to other scores such as 4C and NEWS, CURB-65 has a poor performance with an AUC of 0.80 [26]. However, this was a retrospective single-center study. As for the 4C Score, studied among the Japanese population, it was shown to be a good estimate of mortality and severity of COVID-19 [27]. This was also seen in a study done in Italy by De Vito et al., where the mortality rate went up to 41.7% among the group that was labeled as high risk as per the 4C score [28]. The sensitivity and specificity of the 4C score were acceptable as per the study done by Citu et al., who documented an AUC of 0.81 [26]. Also, a study conducted by Kamran et al. showed that the CALL score had a strong association with predicting progression and mortality in COVID-19 patients [29]. This was also emphasized in a study done by Sanhueza et al, where a 54% increase in 28-day mortality risk was seen with each one-point increase in the CALL score [30].

A retrospective cohort study conducted by Jilanee et al. comparing the performance of several scores (CALL, MuLBSTA, Qsofa, and CURB-65) in predicting mortality in hospitalized patients with COVID-19 showed that the CALL score has a better sensitivity than the other scores [15]. Another prospective study that was done by Yildiz et al. comparing the ability of the following scores to predict critical illness, CURB-65, NEWS2 score, COVID-GRAM, and 4C score, showed that the latter had the highest discrimination for mortality prediction [16]. Moreover, this study showed that the NEWS score performed better in ICU admission prediction [26]. In another retrospective study published in 2023 from KSA, it showed that both CURB-65 and 4C scores had a comparable performance and were considered a good tool in predicting outcome among patients hospitalized with COVID-19 [24].

Need for intubation

Endotracheal intubation preserves oxygenation and ventilation and may prevent aspiration in patients. Dyspnea was significantly associated with the need for intubation. In a study done by Cheng et al., it showed that among COVID-19 patients, a respiratory rate higher than 30/min was statistically significant for needing endotracheal intubation (p < 0.05) [31]. Patients with several comorbidities, especially multiple cardiac diseases, diabetes, and hypertension, showed a significant association with the requirement for intubation. These findings are no different from the literature, which showed that the most common comorbidities in COVID-19 patients requiring intubation were the latter mentioned [32-34]. Although other studies have previously shown an increased risk for intubation in patients with COPD, this was not consistent with our study, and the main risk factors for mechanical ventilation and intubation were metabolic and cardiovascular [33,34]. Also, our analysis showed an association between age and the need for intubation, which can be explained by the low threshold to intubate the elderly population due to their lower tolerance for desaturation. As for the percentage of lung involvement, our results go hand in hand with previous studies, which showed that the percentage of compromised lung volume (%CL) has shown high accuracy in predicting the need for oxygenation support and mechanical ventilation [35].

As for the studied scores, all have shown statistical significance in predicting patients requiring mechanical ventilation and intubation. However, the strength of association and the precision of prediction might vary among the scoring systems. Three of our scores COVIC, COVID-GRAM, and CURB-65 showed high sensitivity of 81%, and the specificity for both COVIC and COVID-GRAM was 60%; however, the specificity of CURB-65 was low, reaching 24% for a score of more than 0.5. In a cohort study conducted by Artero et al., when comparing CURB-65 to other scores such as Pneumonia Severity Index (PSI), it showed a low association with a p-value of 0.346 and an area under the receiver operating characteristic curve (AUROC) of 0.572 (0.553-0.592) [36]. In another study done by Cheng et al., the sensitivity and specificity of CURB-65 in predicting the need for invasive ventilation were found to be 55% and 71%, respectively. This study also showed that when comparing this score to APACHE II, it was found that APACHE II was more accurate in predicting the need for intubation among COVID-19 patients [31]. When looking for the ability of the ISARIC4C score in predicting the need for intubation, a study done by Ocho et al. among the Japanese population showed that this score had an area under the curve (AUC) of 0.78 (95% CI: 0.74-0.81) in assessing mortality and need for mechanical ventilation [27]. Despite the presence of studies that demonstrate the effectiveness of CALL and COVIC score in predicting the progression into severe COVID, our search didn’t find literature regarding their benefit in predicting the need for intubation, which is mainly due to it possibly being outside the scope of the score. Further research and validation studies could help determine the best scoring system or a combination of scores for accurate prediction and management of intubation needs in COVID-19 patients.

ICU admission

ICU admission for COVID-19 becomes necessary when a patient’s condition deteriorates. Both altered mental status and dyspnea were associated with an increased need for ICU admission. This could be hypothesized to be due to the hypercapnia seen in severe ultimately causing alteration in mental status. Furthermore, dyspnea is known to be one of the earliest symptoms of respiratory failure in COVID-19 patients, which further requires ICU admission and mechanical ventilation as discussed previously [37]. Other factors associated with increased risk of ICU admission, a history of pulmonary hypertension, chronic kidney disease, cardiac disease, especially valvopathies, and neurological disease; this has been validated in multiple previous studies [38-40]. As for the percentage of lung involvement, there was a significant association with ICU admission; a >50% lung involvement on admission was found to have worse outcomes [41].

In regard to the scores used to assess outcomes in these patients, we noticed a significant association between ICU stay and the following COVID-19 scores: CURB-65 and CALL-score. The fact that higher scores were associated with longer ICU stays and increased need for ICU admission implies that patients with more severe clinical presentations, as indicated by higher scores, tend to experience a longer recovery period in the ICU and worse outcomes [42]. This could be due to the need for more intensive interventions, such as advanced respiratory support or monitoring of multiple organ systems, in patients with greater clinical complexity [42]. Regarding the COVIC score, there were limited data regarding its use in predicting its ability to determine ICU stay. In a study published in 2021 by Heo et al., this integer-based scoring system was found to be a highly effective instrument to predict those COVID-19 patients who need ICU admission [10]. As for the CALL score, in a study done by Wolfisberg et al., it showed that with a cutoff of >9, this score had a 93.5% sensitivity and 94.9% NPV when it comes to predicting the severity of COVID-19 and need for ICU admission [43]. However, we were not able to find a significant association between COVID-GRAM and ICU admission. One possible explanation is that other factors beyond the scope of these scores, such as individual patient responses to treatment or complications during ICU stay, play a more significant role in determining the duration of ICU care and the need for ICU admission.

In our study, the best performance was seen with the COVID-GRAM score, which was initially used for the prediction of this outcome, showing a higher sensitivity among other scores of 84.6% with a relatively acceptable specificity of 62.5%. Moreover, the CURB-65 score proved to have high sensitivity of 80.8%; however, the specificity was low, reaching only 25% and for a score of more than 0.5, which includes even the low-risk population.

The Pearson correlation coefficient showed a positive linear correlation between the age and all the scores used, most remarkably with the ISARIC4C score. Furthermore, the number of comorbidities showed a moderate positive correlation with the scores. However, there was a minimal correlation between the length of ICU stay and the O2 requirements with the scores we used, proving that there is no strong correlation between the scores and the severity of the disease among the population we studied.

When looking at the comparison between the scores, a retrospective observational study done by Doğanay et al. looked into the prognostic accuracy of the scores CURB-65, ISARIC-4C, and COVID-GRAM in patients hospitalized with COVID-19 and compared them in terms of in-hospital mortality and the need for intensive care prediction [44]. In this study, CURB-65 had a better ability to predict in-hospital mortality than ISARIC-4C and COVID-GRAM, with a Youden’s J index of 0.59, 0.27, and 0.01, respectively. In regards to the prediction of ICU admission, Youden’s J index was also higher for CURB-65, reaching 0.63 compared to an index of 0.26 for ISARIC-4C and 0.01 for COVID-GRAM. Neither result was consistent with the results of our study, where ISARIC-4C performed better in mortality prediction and COVID-GRAM had a better ability to predict the need for critical care.

Another retrospective study published in 2023 studied the predictive value of several scores, including COVID-GRAM, ISARIC-4C, WHO COVID-19 severity classification, CURB-65, and Veterans Health Administration COVID-19 (VACO) index regarding in-hospital mortality, presence of severe or critical illness, need for intensive care treatment, and mechanical ventilation [45]. CURB-65 and ISARIC4C scores performed best in 30 days and in-hospital mortality prediction, which is close to the findings of our study. However, the 4C mortality score and COVID-GRAM were best at predicting the severity of the disease and the critical illness (AUC: 0.785 and 0.717, respectively).

The significance of this study is that the result is extrapolated from local data, which means the possibility of applying it to the Middle East region. It is one of the first publications in the region to compare multiple scores. Another interesting point is that all patients who showed clear signs of ICU admission upon ER arrival were excluded immediately to allow a better assessment of the accuracy of the scores.

Limitations

The data was obtained in 2021, and it is from a single center and is retrospective. Data is susceptible to selection bias due to possible inaccurate data entry in patients' records. With the recent emergence of COVID-19, the profile of this virus is in a continuous state of change, making these data not an accurate reflection of the current situation, but they may aid in building the baseline profile of the disease and seeing its development. Also, the data didn’t include information regarding vaccination status and the type of medication provided, so the impact of the vaccine and treatment on the disease severity and course was not assessed.

Implications

Knowing that this is one of the few studies done in the MENA region to allow the understanding of the accuracy of various COVID scores in allowing patient triage and giving a glimpse into their outcome. Our study provided data regarding the performance of scores such as CALL and COVID-GRAM in certain domains that weren’t seen in previous studies. Also, we were able to obtain information regarding the efficiency of these scores in different concepts, such as mortality, need for mechanical ventilation, and assessing the need for ICU admission. Further multicentric studies are needed to confirm the accuracy of scores such as the CALL and COVID-GRAM in areas that weren’t tackled before.

## Conclusions

With the huge hit that affected the health care sector due to the COVID-19 pandemic, there is a need to have efficient triaging methods to allow optimization of patient care. Our study assessed the predictive accuracy of multiple COVID-19 severity scores, including COVIC, CURB-65, ISARIC4C, COVID-GRAM, and CALL, on patients presenting to the emergency department. While all scores showed significant correlation with mortality, ISARIC4C and COVIC demonstrated superior sensitivity, whereas CURB-65 had lower predictive accuracy. Despite the limitations of our study, it is one of the few studies in the MENA region to observe the performance of these scores. This highlights the need for further studies to be able to fully understand their accuracy in the region for COVID-19 and any future respiratory pandemics. We think that the integration of these scores into clinical decision-making provides further support to the healthcare system, allowing it to achieve a more efficient job and maintain resources in a country limited economically like Lebanon.

## Figures and Tables

**Figure 4:**
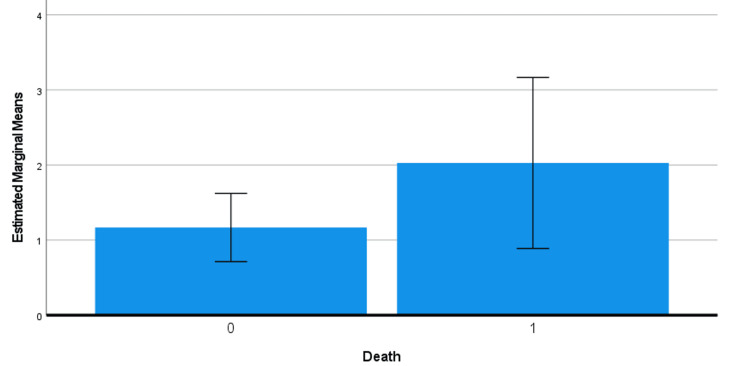
Estimated marginal means of CURB-65 and death Error bars: 95% CI

**Figure 5:**
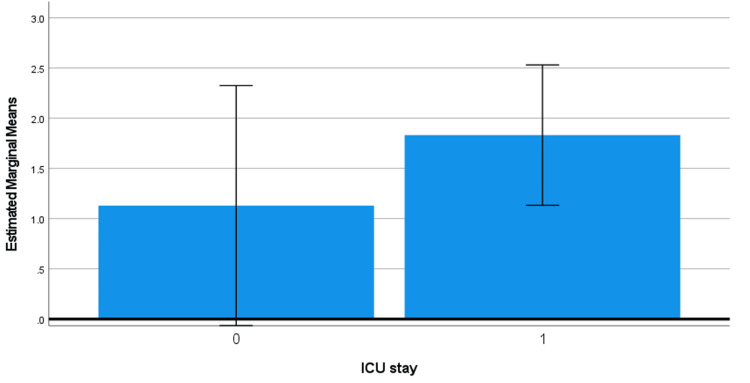
Estimated marginal means of CURB-65 and need for critical care Error bars: 95% CI

**Figure 6:**
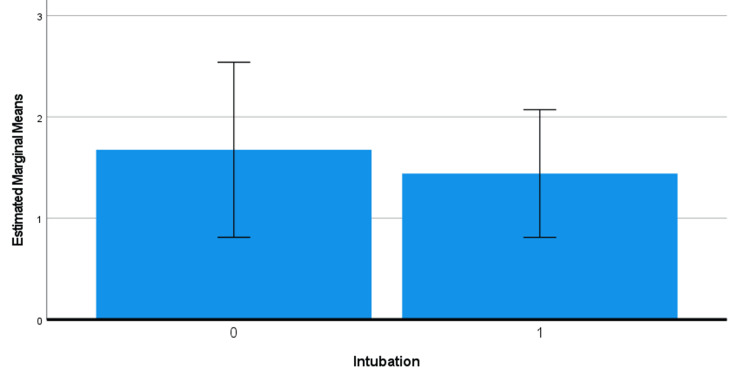
Estimated marginal means of CURB-65 and intubation Error bars: 95% CI

**Figure 7:**
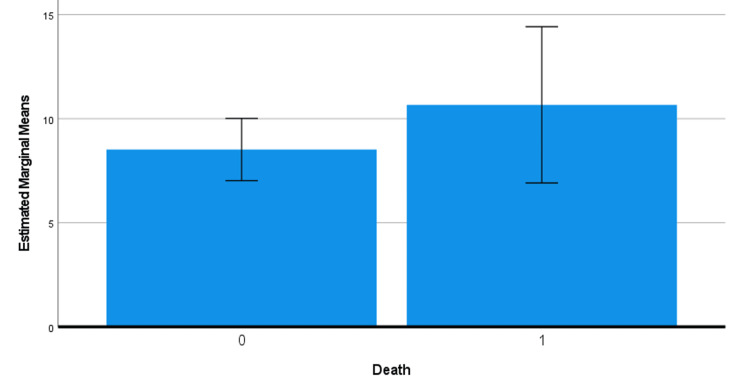
Estimated marginal means of ISARIC4C and death Error bars: 95% CI

**Figure 8:**
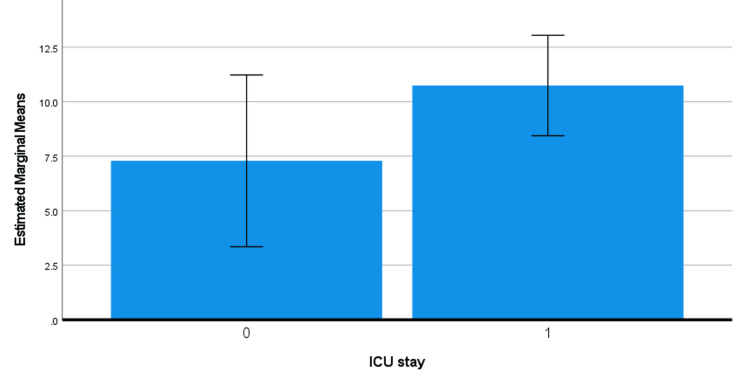
Estimated marginal means of ISARIC4C and critical illness Error bars: 95% CI

**Figure 9:**
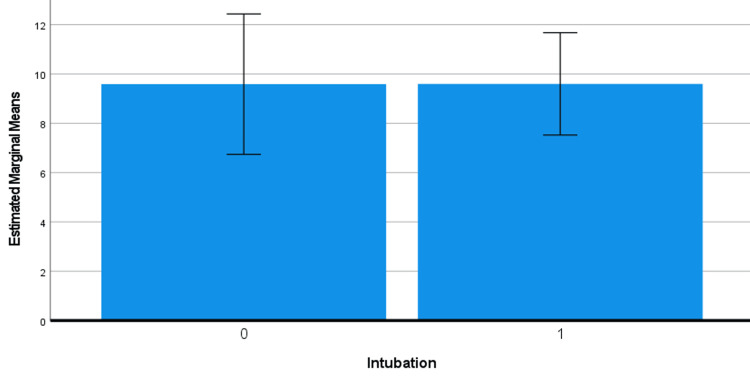
Estimated marginal means of ISARIC4C and intubation Error bars: 95% CI

**Figure 10:**
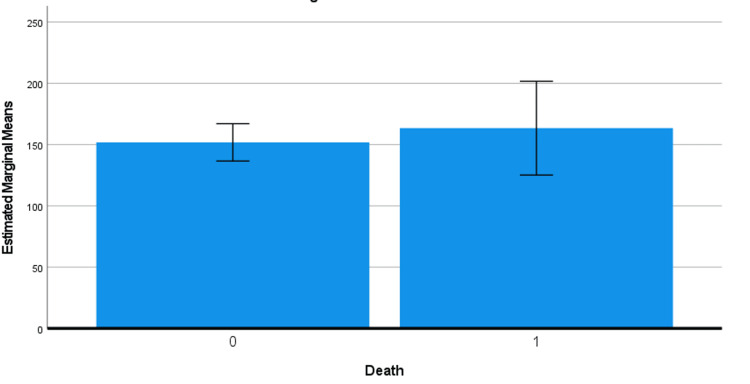
Estimated marginal means of COVID-GRAM and death Error bars: 95% CI

**Figure 11:**
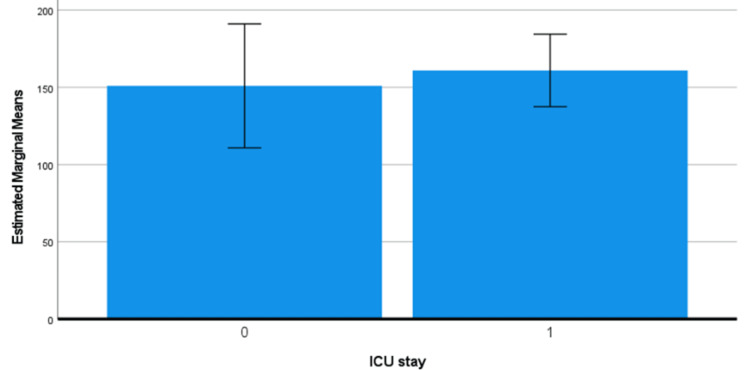
Estimated marginal means of COVID-GRAM and critical illness Error bars: 95% CI

**Figure 12:**
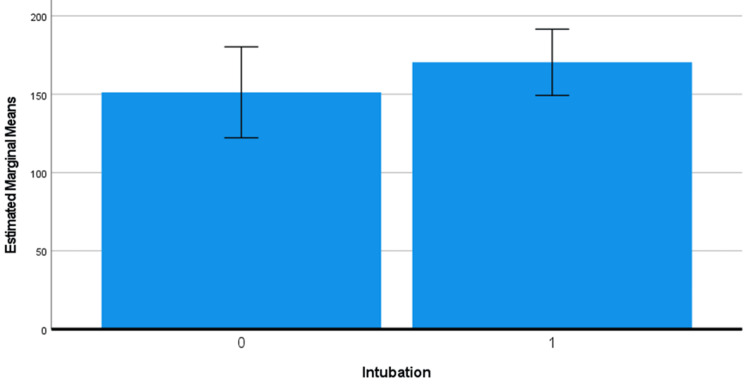
Estimated marginal means of COVID-GRAM and intubation Error bars: 95% CI

**Figure 13:**
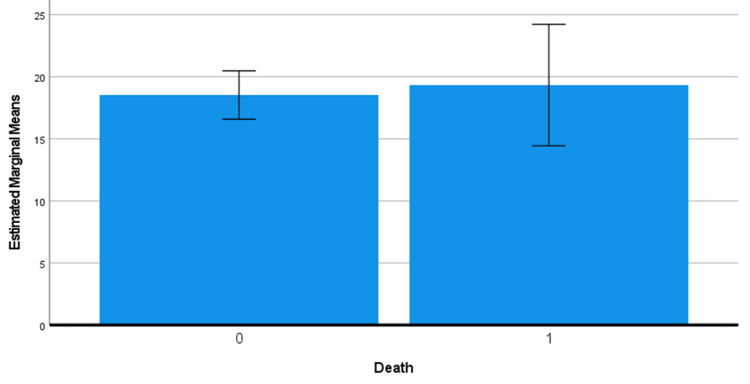
: Estimated marginal means of COVIC and death Error bars: 95% CI

**Figure 14:**
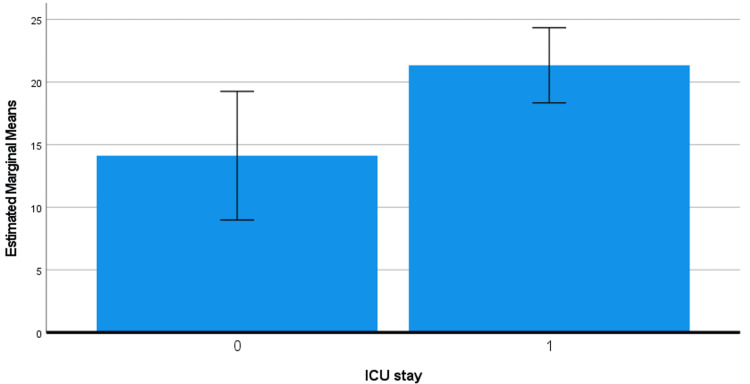
Estimated marginal means of COVIC and critical illness Error bars: 95% CI

**Figure 15:**
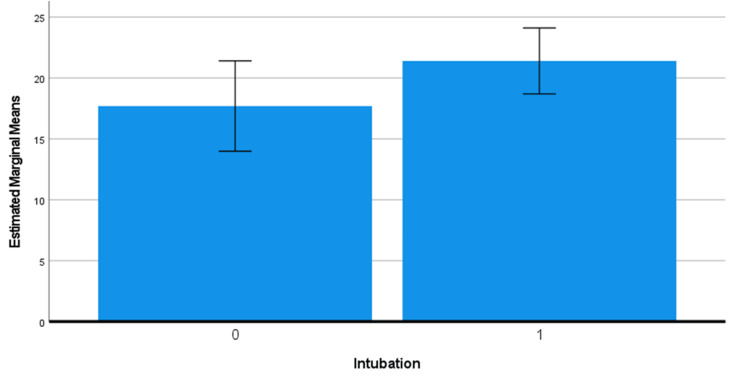
Estimated marginal means of COVIC and intubation Error bars: 95% CI

## Appendices

Find below each score with its variables and its cutoffs. Any additional information can be found in the references attached to this article:

1. CURB-65

This score includes five variables: presence/absence of confusion, urea, respiratory rate of >30 breaths/min, blood pressure (systolic < 90 mmHg or diastolic < 60 mmHg), and age of more than or equal to 65 years. Each one of these gets 1 point to give a total score ranging between 0 and 5. A score higher than 1 puts the patient at an intermediate to high risk for 30-day mortality and suggests the need for in-hospital admission, and if the score is high, it would indicate the need for intensive care admission. 

2. COVID-GRAM

This includes ten variables: age, abnormalities on chest radiography, dyspnea, disturbance in the level of consciousness, number of comorbidities, history of cancer, neutrophil-to-lymphocyte ratio, lactate dehydrogenase (LDH), and direct bilirubin. This score divides the triaged population into low, medium, and high risk. Those with high risk have a more than 40.4% of developing critical illness 

3. COVIC

It includes seven variables: age, gender, initial body temperature on admission, presence of dyspnea, hemoptysis, history of chronic kidney disease, and level of daily living. This score showed an association between the integer-based score and the predicted probability of requiring intensive care.  

4. ISARIC4C

This has eight variables that are assessed upon triaging, which are age, gender, number of comorbidities, respiratory rate, peripheral oxygen saturation, Glasgow Coma Scale, urea, and C-reactive protein (CRP). It is a score from 0 to 21 points; the higher the points, the higher the risk for mortality from COVID. 

5. CALL

This has four components: age, comorbidities (as in the presence of one or more), lymphocyte count, and LDH. This score creates an integer score ranging from 4 to 13 points. A score of 7 and above places the patient in an intermediate to high risk for developing severe COVID-related critical illness. 

Extra tables and figures

**Table 8:** Association between different comorbidities and mortality CXR: chest X-ray; CT: computed tomography; COPD: chronic obstructive pulmonary disease; PHTN: pulmonary hypertension; CKD: chronic kidney disease; CAD: coronary artery disease; CHFpEF: congestive heart failure with preserved ejection fraction; CHFrEF: congestive heart failure with reduced ejection fraction A p-value is considered significant if < 0.05

		Mortality		Chi-square value	p-value
		Yes	No		
Gender	Female	22 (13.3%)	143 (86.7%)	0.90	0.765
	Male	44 (12.4%)	311 (87.6%)		
Symptoms					
Altered mental status	Yes	27 (39.1%)	42 (60.9%)	50.182	<0.001*
	No	39 (8.6%)	412 (91.4%)		
Dyspnea	Yes	47 (14.4%)	279 (85.6%)	2.346	0.126
	No	19 (9.8%)	175 (90.2%)		
Hemoptysis	Yes	0 (0%)	3 (100%)	Fisher	1.000
	No	66 (12.8%)	451 (87.2%)		
Fever	Yes	31 (10.3%)	271 (89.7%)	3.83	0.05*
	No	35 (16.1%)	183 (83.9%)		
Comorbidities					
Diabetes	Yes	26 (16.3%)	134 (83.3%)	2.64	0.104
	No	40 (11.1%)	320 (88.9%)		
Chronic pulmonary disease	COPD	4 (25%)	12 (75%)	Fisher	0.460
	PHTN	0 (0%)	3 (100%)		
	Bronchiectasis	0 (0%)	1 (100%)		
	Asthma	0 (0%)	6 (100%)		
	No	62 (12.6%)	429 (87.4%)		
Renal disease	CKD	6 (33.3%)	12 (66.7%)	Fisher	0.127
	Dialysis	0 (0%)	3 (100%)		
	Transplant	0 (0%)	2 (100%)		
	Others	1 (11.1%)	8 (88.9%)		
	No	59 (12.1%)	429 (87.9%)		
Liver disease	Yes	3 (60%)	2 (40%)	Fisher	0.016*
	No	63 (12.2%)	452 (87.8%)		
Cardiac disease	CAD	13 (19.7%)	53 (80.3%)	Fisher	<0.001*
	CHFpEF	0 (0%)	3 (100%)		
	CHFrEF	1 (50%)	1 (50%)		
	Arrhythmia	2 (25%)	6 (75%)		
	Valvulopathy	12 (30%)	28 (70%)		
	Multiple	66 (12.7%)	454 (87.3%)		
	No	34 (8.7%)	356 (91.3%)		
Neurologic disease	Stroke	0 (0%)	2 (100%)	Fisher	0.074
	Epilepsy	5 (23.8%)	16 (76.2%)		
	Neurological disorders	5 (23.8%)	16 (76.2%)		
	Multiple	0 (0%)	0 (0%)		
	Hydrocephalus	0 (0%)	0 (0%)		
	No	57 (11.8%)	425 (88.2%)		
Malignancy	Solid	6 (22.2%)	21 (77.8%)	Fisher	0.137
	Hematologic	3 (27.3%)	8 (72.2%)		
	Both	0 (0%)	2 (100%)		
	No	57 (11.9%)	423 (88.1%)		
Hypertension	Yes	53 (12.7%)	203 (79.3%)	29.201	<0.001*
	No	13 (4.9%)	251 (95.1%)		

**Table 9:** Association between different variables and the need for intubation CXR: chest X-ray; CT: computed tomography; COPD: chronic obstructive pulmonary disease; PHTN: pulmonary hypertension; CKD: chronic kidney disease; CAD: coronary artery disease; CHFpEF: congestive heart failure with preserved ejection fraction; CHFrEF: congestive heart failure with reduced ejection fraction *= p-value considered significant if < 0.05

				Intubation			Chi-square value	p-value
		Yes			No			
Infiltrates on CXR/CT	Yes		68 (15.1%)		383 (84.9%)		0.582	0.446
	No		8 (11.6%)		61 (88.4%)			
Sex	Female		21 (12.7%)		144 (87.3%)		0.690	0.406
	Male		55 (15.5%)		300 (84.5%)			
Symptoms								
Altered mental status	Yes		32 (46.4%)		37 (53.6%)		64.311	<0.001*
	No		44 (9.8%)		407 (90.2%)			
Dyspnea	Yes		58 (17.8 %)		268 (82.2%)		7.063	0.008*
	No		18 (9.3%)		176 (90.7%)			
Hemoptysis	Yes		0 (0%)		3 (100%)		Fisher	1.000
	No		76 (14.7%)		441 (85.3%)			
Fever	Yes		43 (14.2%)		29 (85.8%)		0.082	0.775
	No		33 (15.1%)		185 (84.9%)			
Comorbidities								
Diabetes	Yes		32 (20%)		128 (80%)		5.37	0.02*
	No		44 (12.2%)		316 (87.8%)			
Chronic pulmonary disease	COPD		4 (25%)		12 (75%)		Fisher	0.380
	PHTN		1 (33.3%)		2 (66.7%)			
	Bronchiectasis		0 (0%)		1 (100%)			
	Asthma		0 (0%)		6 (100%)			
	No		71 (14.5%)		420 (85.5%)			
Renal disease	CKD		6 (33.3%)		12 (66.7%)		Fisher	0.235
	Dialysis		0 (0%)		3 (100%)			
	Transplant		0 (0%)		2 (100%)			
	Others		1 (11.1%)		8 (88.9%)			
	No		69 (14.1%)		419 (85.9%)			
Liver disease	Yes		2 (40%)		3 (60%)		Fisher	0.157
	No		74 (14.4%)		441 (85.6%)			
Cardiac disease	CAD		0 (0%)		3 (100%)		Fisher	0.004*
	CHpEF		1 (50%)		1 (50%)			
	CHrEF		3 (27.3%)		8 (72.2%)			
	Arrhythmia		3 (37.5%)		8 (72.7%)			
	Valvulopathy		3 (37.5%)		5 (62.5%)			
	Multiple		9 (22.5%)		31 (77.5%)			
	No		44 (11.3%)		346 (88.7%)			
Neurologic disease	Stroke		1 (7.7%)		12 (92.3%)		Fisher	0.243
	Epilepsy		0 (0%)		2 (100%)			
	Neurological disorders		5 (23.8%)		16 (76.2%)			
	Multiple		1 (100%)		0 (0%)			
	Hydrocephalus		0 (0%)		1 (100%)			
	No		69 (14.3%)		413 (85.7%)			
Malignancy	Solid		4 (14.8%)		23 (85.2%)		Fisher	0.898
	Hematologic		2 (18.2%)		9 (81.8%)			
	Both		0 (0%)		2 (100%)			
	No		70 (14.6%)		410 (85.4%)			
Hypertension	Yes		58 (22.7%)		198 (77.3%)		26.125	<0.001*
	No		18 (6.8%)		246 (93.2%)			

**Table 10:** Association between different variables and critical care admission CXR: chest X-ray; CT: computed tomography; COPD: chronic obstructive pulmonary disease; PHTN: pulmonary hypertension; CKD: chronic kidney disease; CAD: coronary artery disease; CHFpEF: congestive heart failure with preserved ejection fraction; CHFrEF: congestive heart failure with reduced ejection fraction *= means the correlation is significant at the p < 0.05 level

		ICU stay		Chi-square value	p-value
		Yes	No		
Infiltrates on CXR/CT	Yes	132 (29.3%)	319 (70.7%)	1.086	0.297
	No	16 (23.2%)	53 (78.8%)		
Sex	Female	38 (23%)	127 (77%)	3.502	0.061
	Male	110 (31%)	245 (69%)		
Symptoms					
Altered mental status	Yes	45 (65.2%)	24 (34.8%)	52.787	<0.001*
	No	103 (22.8%)	348 (77.2%)		
Dyspnea	Yes	113 (34.7%)	213 (65.3%)	16.503	<0.001*
	No	35 (18%)	159 (82%)		
Hemoptysis	Yes	0 (0%)	3 (100%)	Fisher	0.562
	No	148 (28.6%)	369 (71.4%)		
Fever	Yes	78 (25.8%)	224 (74.2%)	2.454	0.117
	No	70 (32.1%)	148 (67.9%)		
Comorbidities					
Diabetes	Yes	59 (36.9%)	101 (63.1%)	8.035	0.005*
	No	89 (24.7%)	271(75.3%)		
Chronic pulmonary disease	COPD	9 (56.3%)	7 (43.8%)	Fisher	0.019*
	PHTN	2 (66.7%)	1 (33.3%)		
	Bronchiectasis	0 (0%)	1 (100%)		
	Asthma	0 (0%)	6 (100%)		
	No	137 (27.9%)	354 (72.1%)		
Renal disease	CKD	11 (61.1%)	7 (38.9%)	Fisher	0.027*
	Dialysis	1 (33.3%)	2 (66.7%)		
	Transplant	0 (0%)	2 (100%)		
	Others	2 (22.2%)	7 (77.8%)		
	No	134 (27.5%)	354 (72.5%)		
Liver disease	Yes	4 (80%)	1 (20%)	Fisher	0.025*
	No	144 (28%)	371 (72%)		
Cardiac disease	CAD	24 (36.4%)	42 (63.6%)	Fisher	<0.001*
	CHFpEF	1 (33.3%)	2 (66.7%)		
	CHFrEF	1 (50%)	1 (50%)		
	Arrhythmia	6 (54.5%)	5 (45.5%)		
	Valvulopathy	5 (62.5%)	3 (37.5%)		
	Multiple	22 (55%)	18 (45%)		
	No	89 (22.8%)	301 (77.2%)		
Neurologic disease	Stroke	6 (46.2%)	7 (53.8%)	Fisher	0.007*
	Epilepsy	0 (0%)	2 (100%)		
	Neurological disorders	11 (52.4%)	10 (47.6%)		
	Multiple	1 (100%)	0 (0%)		
	Hydrocephalus	0 (0%)	1 (100%)		
	No	129 (26.8%)	353 (73.2%)		
Malignancy	Solid	7 (25.9%)	20 (74.1%)	Fisher	0.547
	Hematologic	5 (45.5%)	6 (54.4%)		
	Both	0 (0%)	2 (100%)		
	No	136 (28.3%)	344 (71.1%)		
Hypertension	Yes	101 (39.5%)	15 (60.5%)	29.92	<0.001*
	No	47 (17.8%)	217 (82.2%)		

Further figures available regarding the estimated marginal means of CALL score with death, ICU stay, and intubation upon request (couldn't be placed due to the limitation in the number of figures).
